# Evaluation of Stress Accompanying Immunocontraceptive Vaccination in Donkeys

**DOI:** 10.3390/ani12040457

**Published:** 2022-02-13

**Authors:** Erik W. Peterson, Lorenzo G. T. M. Segabinazzi, Robert O. Gilbert, Don R. Bergfelt, Hilari M. French

**Affiliations:** Department of Clinical Sciences, Ross University School of Veterinary Medicine, P.O. Box 334, Basseterre 00334, Saint Kitts and Nevis; lsegabinazzi@rossvet.edu.kn (L.G.T.M.S.); rogilbert@rossvet.edu.kn (R.O.G.); dbergfelt@rossvet.edu.kn (D.R.B.); hfrench@rossu.edu (H.M.F.)

**Keywords:** *Equus asinus*, contraceptive, feral, overpopulation, animal welfare

## Abstract

**Simple Summary:**

The overpopulation of donkeys is of concern worldwide. Some of the concerns associated with uncontrolled donkey populations are the destruction of habitat, competition for feed resources, and negative human–animal interactions. One of the most effective and humane solutions is the use of immunocontraception. Immunocontraception is the use of an animal’s immune system to prevent it from fertilizing offspring by the administration of a vaccine that targets a specific portion of the reproductive system. This study sought to measure and compare the amount of stress that is imposed when two different immunocontraceptives were administered to female donkeys. The results showed that physical exam parameters (temperature, pulse, and respiration) were not affected significantly but the measurement of stress hormones suggests an acute stress response after the first vaccination that was followed by a chronic stress response after a booster vaccination 35 days later, especially in donkeys that had reactions at the injection site. Further studies would be required to identify and reduce the stress associated with the immunocontraception vaccines that were used.

**Abstract:**

The overpopulation of donkeys is recognized as a problem in many parts of the world. The main concerns with uncontrolled donkey populations are habitat degradation and competition for feed resources between donkeys and other species. One of the most effective and humane solutions is the use of immunocontraception. Therefore, this study sought to evaluate the stress imposed by the use of two formulations of a zona pellucida (ZP) vaccine, a recombinant (reZP) and a native porcine (pZP) vaccine, both formulated with a Freund’s adjuvant. The stress was objectively measured using fecal cortisol concentrations and physical examination parameters at fixed points before and after vaccination. We hypothesized that fewer changes in physical exam parameters and lower fecal cortisol concentrations would be stimulated in jennies treated with the reZP vaccine due to the selection of specific proteins. Twenty-five reproductively sound jennies were randomly assigned to reZP (*n* = 9), pZP (*n* = 8) or control (*n* = 8) groups. The vaccines were administered at five-week intervals. Physical exam parameters and body wall thickness of injection sites were recorded for each jenny for four days post-injections. Fecal samples were obtained every other day from day 0 (first vaccination) through day 6 and on days 35 to 41 after booster. Injection site reactions were common in all groups with the reZP and pZP groups being overrepresented. Lameness was observed in the pZP and reZP groups that were affected by injection site reactions and open abscesses. The present study showed an increase in fecal cortisol concentrations within 4 days after the first vaccination with ZP vaccines and, thereafter, a decrease in cortisol 35 days later after the second vaccination, especially in donkeys with open abscesses. Our results suggest that acute stress (increased cortisol) was induced after the first vaccination, and chronic stress (decreased cortisol) occurred thereafter in association with open abscesses. In conclusion, reZP and pZP formulated with Freund’s adjuvant induced local inflammatory reactions with a differential degree of acute and chronic stress in donkeys.

## 1. Introduction

There are an estimated 44 million donkeys (*Equus asinus*) worldwide [1,2]. Many areas, including the USA, Australia, South American countries, and Caribbean islands, have reported overpopulation concerns [3]. As feral populations increase, population management control has become an important subject of concern. Most overpopulated areas have reported issues due to the release of working donkeys to the wild after the industrial revolution [4]. Uncontrolled breeding leads to depletion of food resources, poor body conditions, starvation, and inhumane treatment of those animals. Additionally, there are ecologic concerns as feral donkeys are serious environmental pests, causing erosion, damaging vegetation, and competing for food and water with other native species.

Many population control methods for feral equids have had limited success. Some of these methods include ground shooting, aerial shooting, surgical ovariectomization, surgical castration, chemical castration, surgical vasectomization, chemical vasectomization, hormone manipulation of the reproductive organs, and immunocontraceptives [5]. While some of these methods have more success than others, many are not socially acceptable to the public. Therefore, researchers have shifted to creating a humane approach to population control that would solve the human–animal conflict. One such approach is the use of immunocontraceptives. In a previous study of our group, we proved that the use of a recombinant zona pellucida (reZP) and native porcine zona pellucida (pZP) vaccines are effective to control reproduction in donkeys [6]. With this approach, the donkey’s reproductive cycle is interrupted, not allowing conception, but the donkey can behave closer to normal as compared to sterilization techniques [7].

A challenge in the approach to donkey population control is determining the “harmful” effects of these methods. In our first study, it was observed that there was a high incidence of vaccine-associated side effects (e.g., local swelling, intermittent lameness observed at the walk, and formation of abscesses) [6]. In addition, it is important to note that in comparison to the horse, the donkey demonstrates a more stoic and less reactive behavior to external insults [8]. This stoic nature makes it more challenging for observers to detect signs of pain or distress, and disease. Therefore, the qualitative measurements used to evaluate these measures used in the horse have proven to be unsatisfactory in the donkey [9]. Recent literature has been published on welfare assessments for donkeys in captivity, but none have been validated for the wild population [10].

Quantitative physiological measures, such as cortisol, may prove to be a better analysis in this more stoic equine species. Cortisol has been used as a marker to evaluate stress in horses under many circumstances such as transportation, performance, reproductive examination, welfare evaluation, and surgical procedures [11,12,13,14,15]. The activation of the hypothalamic–pituitary–adrenal (HPA) axis and the consequent production of cortisol has been clearly demonstrated and reflected regarding acute stress [16,17,18], which agrees with the conventional wisdom of acute stress resulting in increased cortisol concentrations. Conversely, in the case of chronic stress, the response of the HPA axis is unclear. Multiple studies have shown a reduction of fecal and plasma cortisol concentrations in animals and humans with a variety of chronic stressors [14,19,20,21,22,23]. Therefore, based on the need of understanding the stress caused by the administration of the reZP and pZP vaccines for population control in donkeys, the aims of this project are: (1) to validate an enzyme immunoassay (EIA) kit for the measurement of fecal cortisol in donkeys; (2) to evaluate and compare injection site reactions, physical parameters of stress, and fecal cortisol concentrations in jennies before and after the administration of Freund’s adjuvant (control), reZP or pZP vaccines. We hypothesize that: (1) the enzyme immunoassay selected for this study can be reliably used to measure fecal cortisol in donkeys; (2) the administration of reZP vaccine will induce fewer injection site reactions and fewer changes in stress parameters in donkeys compared to the pZP vaccine.

## 2. Materials and Methods

This study was performed according to good clinical practice standards at Ross University School of Veterinary Medicine (RUSVM). The study protocol was approved by RUSVM Institutional Animal Care and Use Committee (protocol no. 15.12.032), which is accredited by the Association for Assessment and Accreditation of Laboratory Animal Care International.

### 2.1. Vaccines and Chemicals

The recombinant ZP3 and ZP4 proteins, supplied by the Council for Scientific and Industrial Research, Pretoria, South Africa, were expressed in *E. coli* according to Gupta et al. [24] with several modifications. The reZP vaccine used in the current study comprised recombinant ZP3 and recombinant ZP4, containing the promiscuous T-cell tetanus and bovine RNase epitopes at the N-terminus, respectively. The recombinant ZP3 and ZP4 proteins were analyzed via SDS-PAGE gels and confirmed by LC–MS peptide mapping. ZP3 with the TT epitope had 93.3% coverage at 95% confidence, while ZP4 with the bRNase epitope had 92.6% coverage at 95% confidence, analyzed with ProteinPilot Software (AB Sciex, AB Sciex, Foster City, CA, USA). The resultant reZP product was lyophilized in vials, each containing 2 mg of each protein. Before administration, individual vials were reconstituted with 5 mL sterile water for injection providing a final concentration of 400 µg/mL of each protein. For the primary dose, 0.5 mL of reZP (200 µg per protein) was mixed with 0.5 mL Freund’s complete adjuvant (FCA), whereas for the booster doses, 0.5 mL reZP was mixed with 0.5 mL Freund’s incomplete adjuvant (FIA) [25].

The native pZP was prepared according to a standard method [26] and was supplied lyophilized in 1 mg vials by Trumpeter Farms and Veterinary Service (Winters, CA, USA). Each vial was reconstituted with 5 mL sterile water for injection, providing a final protein concentration of 200 µg/mL. Similarly as for the reZP, the primary dose of pZP (100 µg, 0.5 mL of pZP) was mixed with 0.5 mL FCA and 0.5 mL of FIA was used for the booster. 

An adjuvant control consisted of 0.5 mL sterile saline emulsified with 0.5 mL FCA for the primary dose and 0.5 mL sterile saline emulsified with 0.5 mL FIA for the booster dose. The FCA consisted in mineral oil and a dried inactivated mycobacterium, whereas the FIA consisted in mineral oil only.

### 2.2. Animals and Experimental Design

This study took place at Ross University School of Veterinary Medicine (RUSVM) on the island of St. Kitts in the federation of St Kitts and Nevis. Twenty-five jennies in late term gestation, based upon transabdominal ultrasound, were procured from the island of Nevis. On capture, all appeared to be in good body condition and weighed 100 to 140 kg. No additional history was available. The jennies were subsequently transported to St. Kitts via water ferry. On arrival at RUSVM, all were microchipped and individually identified with numbered collars. Individual ages varied from three to 13 years, estimated according to the appearance of the incisors on dental inspection [27]. The jennies were kept in a grass pasture and supplemented with fresh-cut Guinea grass (*Megathyrsus maximus*) daily, had free access to fresh clean water, and were provided a trace mineral salt block. The jennies were allowed to acclimate to their new environment for ten months to minimize the effects of capture and transportation. During this time, the jennies were trained to lead with a halter and lead rope, and to stand in a set of miniature horse stocks for transrectal ultrasonography. Pelleted horse feed was used as a reward for positive behaviors. The jennies were consistently handled in the presence of the herd. 

The jennies were randomly allocated to three study groups and received the first treatment at day 0 (D0) and a booster 35 days later (D35). Group 1 (*n* = 9), jennies received the reZP vaccine in the complete Freund’s adjuvant (D0) and thereafter one booster vaccine with the incomplete Freund’s (D35). Group 2 (*n* = 8) received the pZP vaccine with the complete Freund’s adjuvant (D0) and one booster with the incomplete Freund’s adjuvant (D35). Group 3 (*n* = 8) received complete Freund’s adjuvant (D0) and second injection with incomplete Freund’s adjuvant (D35) and acted as “controls”.

All treatments were administered intramuscularly by injection into the left and right gluteal muscles (1st and 2nd injections, respectively). Injections were administered using 3 cc Monoject™ syringes with a 18 gauge (Becton Dickinson, Franklin Lakes, NJ, USA) by a 1.5 inch hypodermic needle. The injection sites were clipped with a #40 blade and aseptically prepared with 2% chlorohexadine scrub and 70% isopropyl alcohol prior to administration of the vaccine. 

### 2.3. Physical Examination

Jennies were monitored for five consecutive days following vaccination (day 0, day of vaccination, through day 4). Rectal temperature, pulse, and respiration were accessed and if out of the physiological parameters for donkeys (pulse 36–52 bpm; respiration 12–28 rpm; temperature 97.2–100 °F), it was recorded as an adverse effect [28]. Injection site reactions (swelling or abscesses) and signs of lameness, if noted, were recorded for each jenny, and recorded as an overall finding throughout the experiment time. The abscesses’ content was collected via needle aspiration and submitted for bacterial culture. In the case of open abscesses, the lesion was lavaged with sterile saline solution. The body wall thickness of the injection site was measured in centimeters (cm) via transcutaneous ultrasonography utilizing a FUJIFILM Sonosite, Inc. (Bothell, WA, USA), SonoSite Edge Vet ultrasound with a 10–5 MHz linear transducer. All procedures and physical examinations were performed between 06:00 and 08:00 A.M. each day.

### 2.4. Fecal Sample Collection and Steroid Extraction

Fecal samples were obtained via transrectal palpation once a day during three days before vaccination and every other day from day 0 through day 6 after the first vaccine and day 35 to day 41 (booster, day 35) and stored at −20 °C in airtight polypropylene tubes. Prior to steroid extraction and analysis, fecal samples from each donkey were weighed to 0.1 g, placed in an Eppendorf tube, and lyophilized using a scientific freeze dryer (Harvest Right^®^, Salt Lake City, UT, USA) for 24 h. Freeze-dried samples were pulverized into a powder using a Dounce homogenizer. Initially, 500 μL of 100% ethanol was added to each tube with additional homogenization or mixing for 30 s. A final 500 μL of ethanol was added for a total of one mL and vortexed for 30 min. Subsequent to microcentrifuge at 14,000 rpm for 10 min, 200 μL of supernatant from each tube was transferred into corresponding clean Eppendorf tubes and dried on a SpeedVac^®^ (Thermo Fisher Scientific Inc., Waltham, Massachusetts, MA, USA) for approximately 30 min at 30 °C. Dried fecal extracts were reconstituted with 10 μL ethanol followed by 190 μL assay buffer resulting in 5× concentrated samples. To ensure complete steroid solubility, the samples were vortexed and sonicated for 3 s and 5 min, respectively, and left at room temperature for 5 min until analysis of total cortisol and cortisol-like metabolites using a commercial enzyme-immuno-assay (EIA) kit (Arbor Assay, K003-H5; Ann Arbor, MI, USA) validated for use with donkey feces.

### 2.5. Cortisol Assay Validation and Analysis

Validation (spike/recovery and parallelism) of the EIA kit was conducted using donkey feces collected in the morning and afternoon from control male (*n* = 2) and female (*n* = 2) donkeys. Individual samples were spiked with a known amount of cortisol reference standard (1600 pg/mL) and extracted as previously described. The ratio of observed:expected cortisol reflects the efficiency of the extraction process. Acceptable recovery values range from 80 to 120% [29]. To assess parallelism, a fecal sample collected in the morning from a control male was extracted as described and subsequently diluted (1×, 2×, 4×) with assay buffer and analyzed. Acceptable CVs encompassing the range of dilutions are typically ≤30% [30,31].

### 2.6. Statistical Analysis

The results are reported as the means ± SEM or SD as stipulated. Pearson correlation coefficients were calculated for variables (body wall thickness, cortisol concentration, experimental group). Pre-vaccination concentrations of cortisol were averaged over all groups pre-vaccination (days −3, −2, −1) and used a reference to calculate and analyze the percent change in cortisol on the day of vaccination (day 0) relative to post-vaccination.

The effect of the vaccine on body wall thickness was examined by mixed-effects linear regression, using the experimental group as a fixed variable and jenny as a random variable to account for repeated measures. The relationship between body wall thickness, fecal cortisol difference, and experimental group and day relative to vaccination was explored by mixed-effects multivariate linear regression, with jenny as a random variable to account for repeated measures. A backward elimination procedure was used to derive the best fit model. Injection site reactions and lameness were assessed using Kruskal–Wallis test followed by Dunn’s test. All calculations were made using Stata IC version 15.1 (Stata Corp LLC, College Station, TX, USA). Significance was set at *p* < 0.05.

## 3. Results

### 3.1. EIA Validation

**Figure 1:**
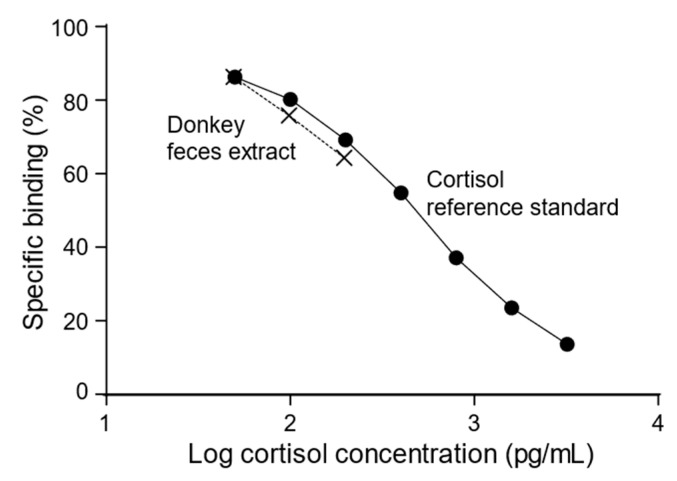
A fecal sample from a control male donkey was extracted and subsequently diluted (1×, 2×, 4×) with assay buffer and analyzed. Visually, various dilutions paralleled the cortisol reference standard curve with an overall CV of 21.2% that encompassed the range of dilutions.

Overall mean recovery of cortisol from spiked fecal samples was 90.8% as shown in Table 1. Visually, various dilutions of a fecal extract paralleled the cortisol reference standard curve with an overall CV of 21.2% that encompassed the range of dilutions as shown in Figure 1. Intra- and inter-assay CVs were 9.8 and 15.7%, respectively, with the lowest detectable concentration at 17.3 pg/mL.

### 3.2. Clinical Parameters, Injection Site Reactions, and Lameness

None of the jennies had changes in the clinical parameters (pulse, respiration, and rectal temperature) post-vaccination. However, injection site reactions were common, and especially so in the experimental groups (reZP and pZP; *p* < 0.05). The injection site reactions after vaccine 1 occurred in 88.9% (8/9) of the reZP group, 75.0% (6/8) of the pZP, and 25.0% (2/8) of the control group. After vaccine 2, 100.0% (9/9) in the reZP group, 75.0% (6/8) in the pZP, and 25.0% (2/8) in the control group had injection site reactions. These reactions consisted of sterile abscesses, confirmed by bacteriological examination. The abscesses that spontaneously ruptured (“open abscess”) occurred 88.9% (8/9) in the reZP group, 50.0% (4/8) in the pZP group, and 12.5% (1/8) in the control group after vaccine 1. After vaccine 2 open abscess occurred in 100% (9/9) in the reZP group, 50% (4/8) in the pZP group, and 0.0% (0/0) in the control group.

**Figure 2:**
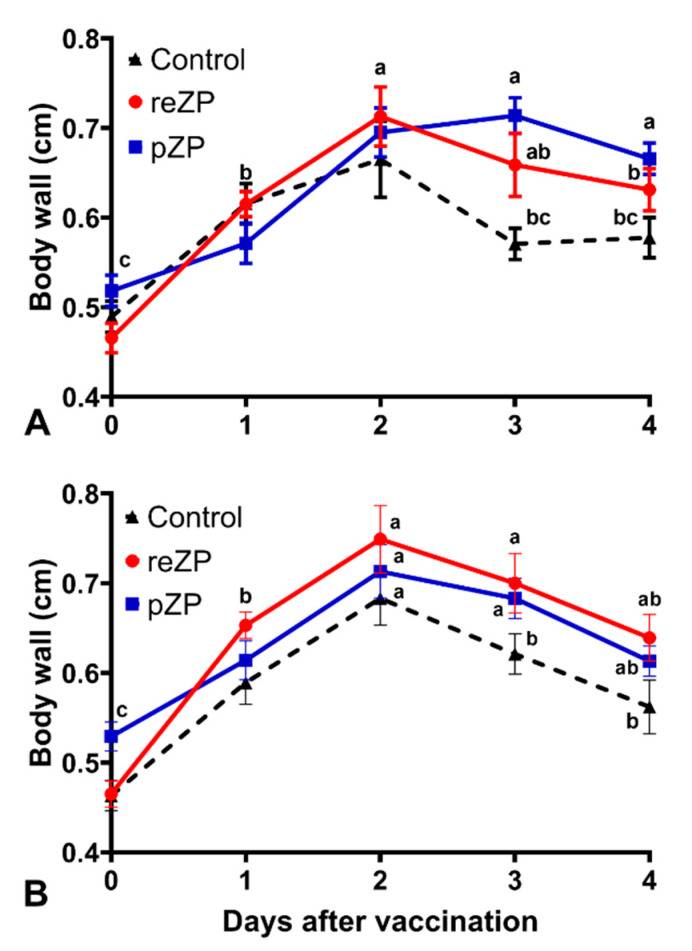
Body wall thickness (cm) of the injection site measured by an ultrasonographic image of donkeys after vaccination with recombinant zona pellucida (reZP, *n* = 9), porcine zona pellucida (pZP, *n* = 8), or Freund’s adjuvant (Control, *n* = 8). (**A**) body wall after the first vaccination. (**B**) Body wall after the booster. Day 0, day of vaccination. Data are expressed as the mean (±SEM). Different superscripts denote effects of time (^a,b,c^) (*p* < 0.05).

Sterile abscessation of injection sites was observed in 9/9 (100%) jennies in the reZP group, 7/8 (87.5%) jennies in the pZP group, and 3/8 (37.5%) jennies in control group from two to 12 weeks post-vaccination. In addition, 3/9 (33%) jennies in the reZP group, 2/8 (25%) jennies in pZP group, and none of the jennies (0/8) in control group presented with signs of lameness and local swelling after vaccine 1. Signs of lameness after vaccine 2 were observed in 3/9 (33%), 3/8 (37.5%), and 0% (0/8) of jennies in reZP, pZP and control groups, respectively. The body wall thickness was not different (*p* > 0.05) between groups in both first and second vaccination (Figure 2). However, day and the interactions between day and treatment were significant in both moments (*p* < 0.02, Figure 2); therefore, the treatments had to stay in the model even though they were themselves not significant overall. The interaction terms arise because the pZP group is lower than the rest on day one but higher on days three and four.

### 3.3. Fecal Cortisol Concentrations

**Figure 3:**
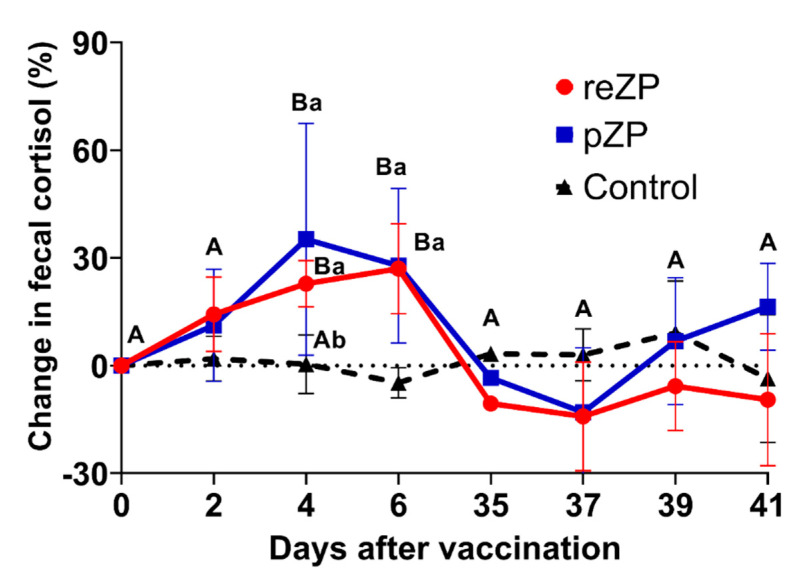
Changes in fecal cortisol concentrations (pg/g) in donkeys after the first vaccination (day 0) and the booster (day 35) with recombinant zona pellucida (reZP, *n* = 9), porcine zona pellucida (pZP, *n* = 8), or Freund’s adjuvant (Control, *n* = 8). Cortisol concentrations from each day were compared with the cortisol concentration at day 0. Different letters denote effects of time (^A,B^) and differences between groups within each time point (^a,b^) (*p* < 0.05).

The intraclass correlation coefficient for fecal cortisol concentrations within jennies was 0.31, indicating a considerable degree of repeatability within jenny. However, concentrations varied widely among jennies. Hence, pre-vaccination concentrations averaged overall for all groups served as a reference on day 0 to evaluate the percent change in concentrations post-vaccination. Mean percent change indicated an increase (*p* < 0.05) in cortisol in the reZP and pZP groups by day 4 with higher (*p* < 0.05) values on days 4 and 6 compared to the control group after the first vaccination (Figure 3). Subsequent to the second vaccination on day 35, mean changes in cortisol remained at pre-vaccination levels for all groups (Figure 3).

**Figure 4:**
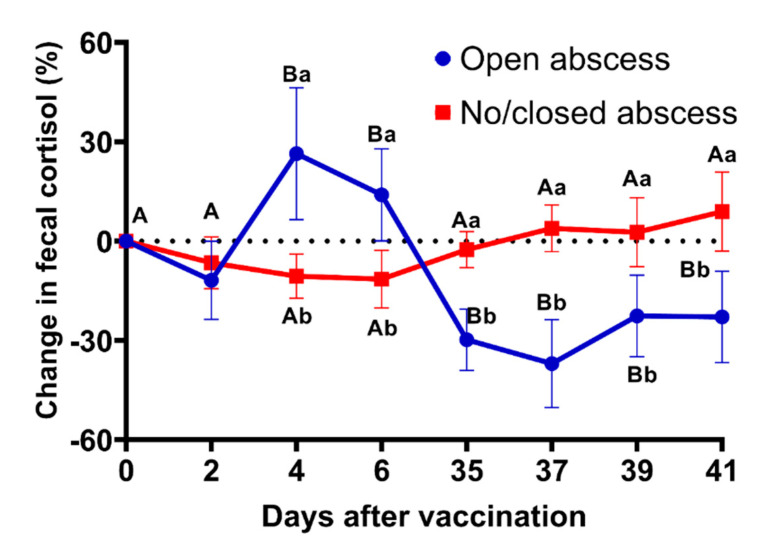
Mean (±SEM) changes in fecal cortisol concentrations (pg/g) in donkeys that did not develop or had a closed abscess after the first (day 0) or second (day 35) injection of zona pellucida vaccine (*n* = 17) or Freund’s adjuvant (*n* = 8). Different letters denote effects of time (^A,B^) and differences between groups within each time point (^a,b^) (*p* < 05).

In association with open vs. no or closed abscesses, mean percent changes in fecal cortisol indicated an increase (*p* < 0.05) by day 4 in jennies with open abscesses after the first vaccination with higher (*p* < 0.05) values on days 4 and 6 compared to jennies with no or closed abscesses (Figure 4). Corresponding to the second vaccination on day 35, mean change in cortisol indicated a decrease (*p* < 0.05) in jennies with open abscesses relative to the first vaccination on day 0 with lower (*p* < 0.05) values on days 35, 37, 39, and 41 compared to jennies with no or closed abscesses (Figure 4).

## 4. Discussion

This study was set forth to evaluate the feasibility and side effects of pZP and reZP vaccines, and Freund’s adjuvant in donkeys. The pZP and reZP vaccines have successfully been used as an immunocontraceptive method in donkeys, reducing pregnancy rates in this species [6]. However, the safety of this method has not been reported in donkeys. Therefore, our study focused on the stress and side effects associated with the vaccination of jennies with pZP, reZP and the vehicle used for the vaccine (Freund’s adjuvant only). For this, physical examination parameters (temperature, pulse rate, and respiratory rate), injection site reactions (swelling or abscesses), body wall thickness at the injection site, signs of lameness, and fecal cortisol concentrations were evaluated pre- and post-vaccination with pZP and reZP vaccines, and in the control group (Freund’s adjuvant only).

The vaccination with reZP, pZP, and the injection of Freund’s adjuvant did not change the physical parameters (pulse, respiratory and rectal temperature) of the donkeys in the present study. Likewise, no heart and respiratory rate elevations were seen in horses vaccinated with reZP and pZP [32,33]. These findings are consistent with post-vaccinal (e.g., attenuated Venezuelan equine encephalomyelitis and inactivated equine herpesvirus) clinical parameters in horses and donkeys [34,35,36,37]. In addition, a previous study has shown that a considerable amount of pain is required to elevate the heart and respiratory rate in horses [38]. However, even though changes in the physical parameters were not observed after vaccination of the donkeys in the present study, the reZP and pZP were associated with a mild degree of discomfort or pain exposed by increased body wall thickness on the injection site, abscesses formation, signs of lameness and changes in fecal cortisol levels. Although clinically veterinarians primarily use heart rate to assess the intensity of pain, multiple papers have shown that there are no significant differences in heart and respiratory rates found in pain groups versus control groups with wound sensitivity or perioperative analgesia [39,40]. In addition, heart and respiratory rates and body temperature are only moderately to weakly correlated with orthopedic pain in horses [38].

Body wall thicknesses, measured ultrasonographically, at the injection sites increased in each treatment group, peaking at day 2 post-vaccination. Previous immunocontraception studies have monitored injection site reactions but have only done so subjectively by observation and/or palpation of the injection sites [32,41]. Multiple other papers document the lack of injection site swelling associated with the administration of various vaccines but were also measured subjectively by observation and palpation [34,37]. In one study investigating ovarian dynamics and injection site reactions associated with administration of ZP and GnRH vaccines in mares, a 3-point scale to assess injection site reactions was used based upon palpation of the site and behavior reaction [32]. In the present study, only mild increases in body wall thickness were observed in the 4 days post-vaccination. Because all three treatment groups were affected with abscesses, this local reaction is likely attributed to the use of Freund’s adjuvant, which has been documented to cause injection site reactions but the reactions were reduced by injecting into the gluteal muscles [42,43]. Future research into this subject could be improved with the addition of a saline or sham injection control group which would not be under the influence of the adjuvant. This would potentially clarify the stressful effects of the adjuvants versus the antigens. However, it was possible to observe that both the pZP and reZP groups were significantly more affected than the control group, which suggests that the pZP and reZP antigens increase the development of the abscesses at the injection sites. In addition, more jennies in the pZP and reZP presented signs of lameness.

Total cortisol and cortisol-like metabolite concentrations in donkey feces were quantitated using a validated commercial EIA kit. The success of the validation was based on acceptable values [29,30] of recovery of known amounts of cortisol in feces (91%) and CV of various dilutions of fecal extracts that paralleled the cortisol reference standard curve (21%). Absolute concentrations were standardized by averaging the pre-vaccination results and then by subtraction of the post-vaccination results. This method allowed for the minimization of variances seen with the unadjusted results. It was interesting that the first vaccination elevated the fecal cortisol concentrations in both pZP and reZP. However, this elevation did not happen after the booster (2nd vaccine) when compared with the pre-vaccination. Although there was no statistical difference, it was possible to visualize lower levels of cortisol after 35 days post-vaccination. This result suggests a stressor process after the first injury (“vaccination”) followed by a decreased cortisol concentration in the face of chronic stress [14,44,45]. Decreased cortisol has been reported in many species subject to a variety of chronic stressors. In cattle, social isolation of calves resulted in lower basal plasma cortisol concentrations compared to calves housed in groups [21]. In sheep experiencing severe chronic lameness, there was evidence of lower plasma cortisol concentrations in comparison to those with mild to no lameness [22]. In humans, teachers with perceived stress or “burnout” and those with back pain showed lower salivary cortisol concentration [23,24]. This phenomenon has also been demonstrated in multiple studies evaluating the potential of compromised welfare in horses [14,44]. One study in thoroughbred racehorses showed lower serum cortisol concentrations after chronic training and racing [44]. These studies support our data in the jennies in the reZP and pZP group with the lowest fecal cortisol concentrations post-vaccination, suggesting that chronic stress was induced by adverse side effects (e.g., local injection reaction, abscess, and lameness) caused by vaccination. 

Similarly, it was possible to observe that after the first vaccination, those that would ultimately become more severely affected with open abscesses showed significantly higher fecal cortisol concentrations compared to those without open abscesses. This suggested that there was acute stress induced, supporting conventional beliefs regarding cortisol release in the face of a stressor, and agrees with previous research in equids exposed to an acute stressor [16]. Conversely, 35 days after the first vaccination followed by the booster, those that ultimately developed open abscesses showed a significant decrease in fecal cortisol concentrations compared to those without open abscesses, which may strengthen our hypothesis. Although the demarcation between acute and chronic stress is subjective and unclear, these results suggested that chronic stress was induced in the more severely affected jennies. These findings are consistent with a previous study evaluating horses under chronic stress due to compromised welfare conditions [14], which suggests that chronic stress was induced after vaccination with pZP and reZP.

In addition, the fecal sampling proved to be a valid method but ideally would have been carried out further on the study timeline to give a broader representation in the case of chronic stress. The measurement of fecal cortisol has gained popularity due to its ability to be collected in a minimally invasive manner [46,47]. An additional benefit to measuring fecal steroids is that unlike serum, plasma, and saliva sampling, which only represents a single point in time, fecal samples demonstrate hormone release over a longer period of time. The results allow for a better estimation of a stressor’s effect over an average time period [48]. Alternatively, hair cortisol could be considered to evaluate cortisol concentrations over a prolonged period of time and salivary cortisol could be considered to evaluate the acute stress response. Finally, as previously discussed, it is important to critically interpret cortisol concentrations along with other physiological and physical parameters that may be associated with stress. Evaluation of the combination of data can potentially provide a more accurate interpretation of results, especially in differentiating temporally acute versus chronic stress post-vaccination. The addition of one or both of the Facial Expression Pain Scales in donkeys that have been recently validated [49,50] would be beneficial in the assessment of the jennies’ stress after vaccination. These data could potentially add critical correlations along with the physical examination parameters and cortisol concentrations to assess the amount of stress that is being introduced.

## 5. Conclusions

In conclusion, reZP and pZP formulated with Freund’s adjuvant induce local inflammatory reaction and a certain degree of stress, revealing changes in cortisol levels in donkeys after vaccination. Although reZP induced stress, the differences observed in the present study were not sufficient to recommend one over the other. In addition, fecal cortisol concentrations can be used as an acceptable method of measuring fecal cortisol concentrations in donkeys for future studies.

## Figures and Tables

**Table 1:** Individual donkey fecal samples spiked with a known amount of cortisol reference standard (1600 pg/mL) and extracted. The ratio of observed:expected cortisol reflects the efficiency of the extraction process to recover cortisol and cortisol-like metabolites from feces.

Donkey Sample	Observed (pg/g)	Expected (pg/g)	Recovery (%)
Male 1, a.m.	61.8	76.2	81.1
Male 2, p.m.	76.0	76.2	99.7
Female 1, a.m.	50.1	76.2	65.7
Female 2, p.m.	88.9	76.2	116.7
Mean	69.2	76.2	90.8

## Data Availability

The original contributions presented in this study are included in this article, further inquiries can be directed to the corresponding author/s.
